# Organizational and pedagogical characterization of vocational training in nursing in the Federal Education Network

**DOI:** 10.1590/0034-7167-2025-0144

**Published:** 2026-06-12

**Authors:** Ludmila Anjos de Jesus, Gilberto Tadeu Reis da Silva, Silvana Lima Vieira, Vânia Marli Schubert Backes

**Affiliations:** I Universidade Federal da Bahia. Salvador, Bahia, Brazil; II Universidade do Estado da Bahia. Salvador, Bahia, Brazil; III Universidade Federal de Santa Catarina. Florianópolis, Santa Catarina, Brazil

**Keywords:** Education Nursing Associate, Professional Training, Education Nursing, Health Human Resource Training, Nursing Education Research., Graduación en Auxiliar de Enfermería, Capacitación Profesional, Educación en Enfermería, Capacitación de Recursos Humanos en Salud, Investigación en Educación de Enfermería.

## Abstract

**Objectives:**

to understand the organizational and pedagogical characteristics of mid-level vocational training courses in nursing offered by the Federal Network of Professional, Scientific, and Technological Education.

**Methods:**

this is a descriptive-exploratory research study with a qualitative approach and documentary research. Course Pedagogical Projects were used as data sources, employing structured outlines. The data were subjected to content analysis.

**Results:**

twenty-six projects were analyzed. Converging aspects were identified regarding the intention of providing quality training, socially embedded, and valuing active methodologies. Weaknesses include discrepancies in meeting project composition requirements, mismatches between training objectives and practical experience, inconsistencies in internships, and an incipient focus on the Brazilian Health System.

**Final Considerations:**

the courses carry structural and organizational specificities, raising the challenge of fostering adjustments and updates to qualify training, evoking the strategic role of the Network in transforming power into effective action.

## RESUMEN

**Objetivos:**

comprender las características organizativas y pedagógicas de los cursos de formación técnica de nivel medio en enfermería que ofrece la Red Federal de Educación Profesional, Científica y Tecnológica.

**Métodos:**

investigación descriptiva-exploratoria con enfoque cualitativo y carácter documental. La fuente de datos fueron los Proyectos Pedagógicos de los Cursos, utilizando directrices estructuradas. Los datos se sometieron a análisis de contenido.

**Resultados:**

se analizaron 26 proyectos. Se identificaron aspectos convergentes en la intención de una educación de calidad, socialmente integrada y que valore las metodologías activas. Las debilidades incluyen discrepancias en el cumplimiento de los requisitos del proyecto, desajustes entre los objetivos de la formación y la experiencia práctica, inconsistencias en las prácticas y un enfoque deficiente en el Sistema Único de Salud.

**Consideraciones Finales:**

los cursos presentan especificidades estructurales y organizativas, lo que plantea el reto de impulsar ajustes y actualizaciones para mejorar la formación, destacando el papel estratégico de la Red para transformar el potencial en acciones efectivas.

## INTRODUCTION

Health education presents a historical and ongoing challenge, as health needs continually transform and expand in line with changes in sociopolitical and cultural contexts, requiring, in parallel, ongoing reassessments and reorientations of educational practices. From this perspective, it is emphasized that professional education refers to processes aimed at acquiring knowledge and skills for the exercise of a trade, occupation, or profession, favoring individuals’ rapid insertion into the job market^(1,2)^.

Historically, it was shaped by structural duality, which established distinct training paths according to social class, resulting in educational activities focused on intellectual functions for the socioeconomically more advantaged classes and instrumental-procedural preparation for the less advantaged classes, as they considered manual labor to have no social merit^(3)^. Thus, traditionally, vocational training (VT) was delegated to the socioeconomically less advantaged classes, with intense encouragement to do so over the capacity for criticism of content^(4)^.

In this context, secondary level professional education was outlined with notable highlights of hegemonic groups, characterized by an emphasis on content and restriction on the operationalization of procedures^(2)^. This hegemonic ideology was also reflected in the health sector, especially in VT courses in nursing, which express political and socioeconomic issues in their history.

The VT course in nursing in Brazil emerged in 1966, driven by the insufficient number of nurses to meet the population’s and market’s needs^(5)^. In the current model of healthcare organization, nursing technicians (NTs) perform a large part of the direct patient care activities, in addition to representing the largest workforce in health, corresponding to 59.98% of nursing professionals^(6)^. Therefore, it requires consistent training, with a solid structure based on skills and competencies to meet the population’s health needs with quality, in accordance with the Brazilian Health System (In Portuguese, *Sistema Único de Saúde* - SUS)^(7)^.

However, with regard to training directives, it appears that educational practice has been guided by the Law of Guidelines and Bases of National Education (In Portuguese*, Lei de Diretrizes e Bases da Educação Nacional* - LDB) and by Resolution 1 of the Brazilian National Education Council, which determines the national curricular guidelines for professional and technological education, guided by the Law of Professional Practice, due to the historical gap in specific guidelines^(8)^. It is worth noting that, although these regulations advocate the comprehensive education of students and the development of socio-affective, psychomotor, and cognitive skills, aiming to prepare individuals for the full exercise of citizenship in the world of work and in social practice^(9,10)^, there are still schools where the interest in meeting the labor market’s specific demands predominates, especially in light of the process of commodification of education^(4)^.

In this regard, antagonizing the strictly instrumental vision of professional education, the implementation of the Federal Network of Professional, Scientific, and Technological Education, in 2008, sought to interrupt the VT’s logic in the service of capitalist accumulation, envisioning, in educational work, an important instrument of social policy, generator of opportunities and reducer of inequalities^(11)^.

However, in this scenario, despite the federal government’s organizing role in the training of human resources in the health area, as recommended by Law 8,080^(12)^, the predominance of the private sector in the concentration of mid-level VT in nursing is still noticeable^(13)^, which emphasizes the Network’s potential role in transforming this reality in light of its social function. Therefore, reflections on how VT at the nursing level occurs in this space are important.

Hence, the following research question was outlined: how does vocational professional training in nursing offered by the Federal Network of Professional, Scientific, and Technological Education occur?

Considering that the educational path strongly impacts the professional profile and that training is characterized by a complex and dynamic process, this topic became relevant, given the growing demands on professional competencies and the increasingly pressing need for critical professionals. Furthermore, this research was developed in a priority area of the Ministry of Health, related to “work management and health education”^(14)^, enabling dialogue between health and education policies.

As it consists of a little-researched topic, given the scarcity of existing studies^(15)^, while reiterating the proposal’s originality and novelty, it is expected to contribute to the expansion of this area of knowledge, promote new studies and favor the understanding of elements to support reflections and adjustments aimed at improving training processes.

### Study relevance

This article is the product of a master’s dissertation entitled “*Formação Profissional Técnica em Enfermagem na Rede Federal de Educação Profissional, Científica e Tecnológica*”^(16)^, deposited in the *Universidade Federal da Bahia*’s institutional repository (https://www.repositorio.ufba.br/handle/ri/37326).

## OBJECTIVES

To understand the organizational and pedagogical characteristics of mid-level VT courses in nursing offered by the Federal Network of Professional, Scientific, and Technological Education.

## METHODS

### Ethical aspects

Since this is a documentary study based on publicly available materials available on institutional websites, submission to a Research Ethics Committee was waived. However, the researchers ensured all ethical considerations. To ensure anonymity regarding the Pedagogical Projects’ originating institutions, each project was assigned a code consisting of an acronym, known as CCP, which stands for Course Pedagogical Project, followed by an Arabic numeral, such as CCP1.

### Study design

This is descriptive research of an exploratory nature, with a qualitative approach and documentary nature.

### Study setting

This research presents as a scenario the Brazilian Federal Network of Professional, Scientific, and Technological Education, composed of higher, basic and professional education institutions, multicurricular and multicampus^(11)^. In this study, institutions and their respective teaching units that offer the vocational course in nursing in any modality were selected.

### Data source

Course Pedagogical Projects (CCPs) were used as data sources. It is important to note that a CCP is an official document that presents the concepts and intentions of the archetype that is intended to be formed, revealing the contours of the formative process^(17)^.

### Data collection and organization

To collect data related to the offering of courses, the *Plataforma Nilo Peçanha* (PNP) was used, which is a virtual environment for collecting, validating, and disseminating official statistics from the Federal Network of Professional, Scientific, and Technological Education^(18)^. Through the PNP, it was possible to identify institutions and academic units that offer vocational courses in nursing.

Subsequently, we visited the official portals of each institution, specifically each tab corresponding to the academic unit offering the NT program to obtain the CCP. It is important to note that, as these are multi-campus institutions, a single institution may offer the NT program at one academic unit or campus but not at other units. Due to administrative and pedagogical autonomy, courses offered at different academic units within the same institution require different CCPs.

Institutions that, despite offering a vocational course in nursing, did not have CCPs available on their institutional websites or official portals were excluded.

To support data collection, four structured scripts developed with the help of Microsoft Excel^®^ were used, comprising items related to the issues of interest, as shown in Chart 1. Data collection took place, through indirect documentation, from January to July 2022. Two researchers were involved in data collection, both with previous research experience.

**Chart 1:** Synthetic representation of the issues of interest present in structured scripts, Salvador, Bahia, Brazil, 2025

Script identification	Questions of interest
Script I	Institutions offering the course Location Number of nursing technician courses offered
Script II	Course modality Teaching modality
Script III	Year the Pedagogical Project was developed Pedagogical Project development team
Script IV	Course identification Course justification Objectives Requirements and access methods Professional completion profile Curriculum organization Learning assessment criteria and procedures Available infrastructure Professor and administrative staff qualification profile Certificates and diplomas to be issued Deadline for course completion Identification of mandatory supervised internship activities

### Data analysis

This study comprised the vocational courses in nursing offered by the Federal Network of Professional, Scientific, and Technological Education, regardless of the offering modality, which made their CCPs available on the institutions’ official websites.

The data were organized in a database created using Microsoft Excel^®^. Content analysis was used for analysis^(19)^. Thus, initially, pre-analysis was carried out, in which the first contact with the field of the research *corpus* occurred, through material skimming and organization, followed by material exploration, for subsequent treatment of results, through interpretations and inferences^(19)^. The data were organized according to thematic similarity for later interpretation and inferences. To ensure the study validity, the data obtained were discussed in light of national legislation and guidelines, and Federal Network of Professional, Scientific, and Technological Education concepts.

## RESULTS

### Organizational characterization of vocational courses in nursing offered by the Federal Network of Professional, Scientific, and Technological Education

Of the 48 vocational programs in nursing offered by the Federal Network of Professional, Scientific, and Technological Education, only 26 CCPs were located on the institutions’ official websites. Concerning the publication year of these CCPs, it was observed that it varied from 2010 to 2022. Among the professionals involved in CCP design, professors predominate, having participated in the development of 12 Pedagogical Projects, and only two indicate the presence of student representatives on the development team. In relation to the course modality, the subsequent format predominates, revealed in 25 documents.

All identified CCPs demonstrate the rationale for offering the course. The motivation for offering NT courses within the Federal Network of Professional, Scientific, and Technological Education is evident in the commitment to meeting social needs, especially those related to the shortage of professionals with these qualifications. CCPs agree on the need to contribute to qualified and socially integrated training, aimed at meeting local and regional health demands, in addition to promoting the expansion of NT course offerings in the public sector, with the aim of improving the population’s health and quality of life.

[...] *the offer of a vocational course in nursing is justified* [...]*, responding to a demand from the municipality and region itself* [...]. (CCP19)

[...] *lack of public vocational training courses in the health sector, with the training demands of workers in this area being met almost exclusively by the private sector* [...]. (CCP22)

Despite the affluence and concern for social demands, only three CCPs emphasize citizenship-oriented training, and only four indicate alignment with the SUS concepts. In relation to the general objectives, presented in all CCPs, there is general agreement on the purpose of training qualified professionals with solid vocational and scientific skills capable of contributing to improvements in healthcare and society.

*To train qualified professional-citizens to act and intervene in the world of work, developing activities in different nursing team care scenarios, with an emphasis on health promotion* [...]. (CCP1)

[...] *the region is lacking qualified labor, and the training of these professionals will provide the community with an improvement in its quality of life* [...]. (CCP15)

In general, the highlighted CCPs contain the minimum requirements for their composition. For some elements, particularly certificates and diplomas to be issued (CCP2 and CCP10) and admission requirements and forms (CCP10 and CCP21), only the internal regulatory standards were mentioned, without presenting the data.

All CCPs reveal the professional profile upon completion of the course. In this context, we observed a focus on the archetype of a critical, reflective NT capable of effecting change in their environment, with the ability to work in a variety of settings, as expressed in the excerpt below:

[...] *must have the ability to develop activities inherent to their qualification, promote quality humanized care, capable of understanding the health-disease process in its entirety, acting in a reflective, critical and creative way with the objective of meeting the client’s basic needs.* (CCP6)

It was found that the intention of a professional profile aligned with the SUS appeared tentatively in CCPs, as only seven projects followed this perspective. Compliance with the regulations that guide NT training and practice, such as the current Brazilian National Catalog of Vocational Courses (In Portuguese, *Catálogo Nacional dos Cursos Técnicos* - CNCT) and the Professional Practice Law, was also evident.

[...] *must understand the Brazilian Health System principles, valuing comprehensiveness and the individual’s right to assistance at any level of healthcare* [...]. (CCP13)

*The professional profile of graduates of the nursing technician course is based on the Nursing Professional Practice Law 7,498/86* [...]. (CCP21)

Concerning infrastructure, sensitive points were observed regarding the presentation of data and resources available for offering the vocational course in nursing in the Network, highlighting the absence of laboratories and the offering of courses under provisional conditions.

*The infrastructure specified in the CCP regarding the nursing laboratory will cover the specific requirements when the campus is located at the definitive headquarters* [...]. (CCP1)

Although most CCPs present the qualification profile of their staff, gaps in the qualification profile of professors and technical-administrative staff were observed due to omissions or deficiencies in detailed data descriptions, particularly regarding the technical-administrative staff. As for the qualifications of professors, master’s and doctoral degrees predominate.

### Pedagogical characterization of vocational courses in nursing offered by the Federal Network of Professional, Scientific, and Technological Education

Among the items related to pedagogical nuances, weaknesses were identified regarding the lack of syllabi. Regarding learning assessment criteria, continuous, formative, and cumulative processes predominated, with a focus on qualitative aspects.

*The assessment of student achievement must be formative, therefore, comprehensive, procedural and continuous* [...]. (CCP21)

The maximum timeframe for course completion was the most deficient, as nine CCPs failed to address this point. Projects that addressed this topic indicated a range of two to six years as the maximum timeframe for course completion. Regarding the issuance of certification documents after course completion, diplomas predominated.

All CCPs included the curricular organizations employed, with evident diversification in the matrix structures, with a predominance of organization into disciplines and curricular components. It is noteworthy that three CCPs (CCP7, CCP8, and CCP12) did not disclose the bibliographies recommended for the course. However, of the 15 CCPs that explained the methodologies underlying the curricular organizations and guiding the teaching process, there was convergence around problematizing and interdisciplinary concepts, with a focus on active methodologies, integration, and human development.

*The nursing technician course adopts teaching methodologies that encourage dialogue, questioning, interaction and creativity* [...]. (CCP12)

[...] *are didactic-pedagogical strategies adopted. The articulation and interdisciplinarity between the curricular components and the integrative methodologies* [...]. (CCP15)

CCPs include a description of the total course load (tCL) and the distributed course load, with a noticeable variation from 1,440 to 2,724 hours in the tCL, with an average of 1,754 hours. Regarding the distribution of the course load, there was a fluctuation from 1,200 to 2,160 hours, related to theoretical-practical activities, and from 260 to 720 hours, related to the supervised internship.

Concerning the supervised internship, it was observed that all Pedagogical Projects addressed the topic, but only CCP4 specifies the minimum activities to be performed during the internship. There was significant heterogeneity regarding internship timing throughout the course and whether it occurred integrated with curricular components or independently. Some CCPs expressed the internship as congeneric with practices of curricular components and laboratory. Regarding the fields of practice, the hospital context was predominant.

## DISCUSSION

The CCP consists of a basic document that directs the development of educational processes^(20)^. Since it is a guiding document for training, it is recommended that it be public in nature, especially when it comes to government institutions. Thus, the limited number of CCPs available on official portals highlights weaknesses in the principle of transparency of public agencies. Furthermore, it hinders society’s connection to the educational premises and purposes outlined by institutions, which undermines the dynamic interaction between society and schools. It was noted that most CCPs had a considerable time lag and, therefore, are classified as outdated. It is emphasized that institutions are responsible for reviewing their Pedagogical Projects to maintain them as an up-to-date reference instrument compatible with real needs^(10)^.

Concerning the weaknesses evidenced in project construction, it is emphasized that, based on the democratic management of educational processes established by the LDB, it is inferred that CCP elaboration must be guided by the pluralism of ideas, encompassing the school and local community beyond the teaching scope^(9)^. The practice of constructing a CCP must be guided by dialogue between all school stakeholders so that the document reflects the community’s desires and ideals^(21)^.

Regarding the predominance of the subsequent modality, it is inferred that it results from the need to aggregate students, especially those in conditions of greater socioeconomic vulnerability, who need to be inserted into the job market during the opposite shift. Furthermore, it is worth noting that these courses also generate lower costs for institutions. However, it is also worth noting that the Federal Network of Professional, Scientific, and Technological Education prioritizes vocational courses, especially in integrated formats^(22)^.

The identified CCPs meet the requirements regarding the presentation of justifications and course objectives^(10)^. This data demonstrates, in addition to compliance with regulations, a means of clarifying the training’s intention, offering the community parameters that guide and support the course offering.

The justifications touch on nuances alluding to social needs, which reinforces the Federal Network of Professional, Scientific, and Technological Education’s commitment to society and local and regional development, in addition to interfering with the predominant offering of NT courses by the private sector. It is worth noting that the private sector’s hegemony in offering NT courses^(23)^ often results in weakened training tied to market interests. Thus, the Federal Network of Professional, Scientific, and Technological Education positions itself as a project with the potential to reshape this landscape. However, it is considered that the offering of NT courses remains limited given its scope^(24)^. Therefore, despite the demand, it is assumed that the course is not considered a priority in the Network^(23,24)^, which could enrich contributions to society.

On the other hand, although the justifications for the courses are based on social demands, few CCPs emphasize training focused on the exercise of citizenship. Thus, considering the Network’s core and incipient emphasis on citizenship, the need arises to bring CCPs closer to civic and emancipatory education, given that education is an indispensable element for citizenship construction and exercise. Citizenship education, in addition to gaining greater visibility in the Pedagogical Projects of the Network’s vocational courses in nursing, needs to be integrated into the educational process so that it does not become a superficial and empty discourse.

Concerning the justifications and objectives, it was clear that there was little evidence of consonance between the training offered by the Network and the SUS principles. This is contradictory, given that training for the SUS is integrated with social and health demands. In this context, it is clear that training must be aligned with the SUS, aiming to effectively respond to health needs in various health-disease scenarios^(25,26)^. It is worth noting that the Network, because it is located within the federal public sphere, has an even more pressing commitment to training for the SUS. In this regard, in 2024, the Guidelines and Orientations for Vocational training in Nursing were published, which advocates for the organization of training along structural axes aimed at developing competencies for work within the SUS^(27)^.

This study demonstrated that the Federal Network of Professional, Scientific, and Technological Education aims to develop NTs capable of performing the activities inherent to their qualification, and also critically, ethically, and qualified to be agents of transformation in their reality, with several possibilities in fields of activity. Compliance with regulations that guide NT training and practice, such as the CNCT and the Professional Practice Law, was also demonstrated. The data obtained revealed a correlation between the professional profile of NT graduates and the development of competencies involving the knowledge dimensions (learning to know, learning to do, learning to be, and learning to live together), similar to another study^(21)^.

The lack of nursing laboratories and provisional provision, observed in some CCPs, deviates from the requirements and may complicate training. However, it is worth reflecting on the reasons that may contribute to non-compliance with requirements, especially regarding the financial and structural issues of public institutions.

Data deficiencies regarding the team profile, especially technical-administrative staff, contribute to the invisibility of professionals who contribute to the conditions and resources inherent to the training dynamics, even outside the classroom^(28)^. The hegemony of professors with master’s and doctoral degrees presupposes a better training profile and is in line with the LDB, which defends, as pathways to professor qualification, master’s and doctoral courses^(9)^.

Weaknesses related to the lack of syllabi and the indication of time required to complete the course hinder the understanding of the training path. Moreover, the suppression of bibliographies is a violation of legal requirements and hinders the access and understanding of the scientific and theoretical foundations that guide training^(10)^. Learning assessment is presented in accordance with the LDB^(9)^. Therefore, assessment, valued as procedural, requires intentionality and is closely interconnected with the teaching-learning system, and should be transversal to training, distancing itself from specific, isolated, authoritarian and, often, punitive practices.

Regarding curricular organization, it is important to emphasize that disciplines and curricular components express distinct interpretations. Curricular components encompass all the elements that make up curricular integration, whether theoretical or practical. Disciplines, on the other hand, consist of a systematically organized set of knowledge about a subject and represent a type of curricular component^(29)^. It is worth noting that curricula, when organized by disciplines, often segment the content into separate and decontextualized chunks, with isolated basic and clinical chunks being common in the health area, which is worrying, especially in NT training^(30)^.

Based on the considerable number of curricula structured around disciplines, it can be inferred that, when not associated with effective integration strategies, they perpetuate traditional models marked by content fragmentation, which diverge from the educational concepts of the Federal Network of Professional, Scientific, and Technological Education. However, even in this area, when analyzing the methodologies adopted, those with problematizing and interdisciplinary characteristics, with an emphasis on active methodologies, were prominent, converging with the principles of training within the Federal Network of Professional, Scientific, and Technological Education. Such methodologies present themselves as the antithesis of disciplinary and fragmented knowledge. Thus, it can be inferred from these findings that problematization and interdisciplinarity translate into initiatives aimed at bridging the gap in the face of the hegemony of disciplinary-focused curricula.

As for course workload, a significant polarization was observed. All courses adhere to the established minimum theoretical workload of 1,200 hours, and most courses anchor the theoretical-practical workload to the minimum set by the CNCT^(31)^. However, critical weaknesses were highlighted regarding the internship’s workload, favored by the lack of a minimum workload determination for its completion^(31)^. However, given the vacuum caused by the absence of a regulation that clearly defines the minimum workload required for the internship in VT in nursing, Opinion 1/2019 of the Federal Nursing Council recommends the adoption of at least 400 hours for the curricular internship for vocational qualification in nursing in the country^(32)^. It is worth noting that the historical absence of specific directives for VT in nursing has provided room for a heterogeneous scenario, with concrete possibilities of affecting students’ adequate development to achieve the desired profile, especially when they admit restrictions related to students’ contact with real work situations.

Some documents observed that discipline practices were equated with internships, even leading to laboratory practice being counted as supervised internship activities. This finding demonstrates a significant weakness in the training process within the Network, given that exposure to real-world work situations is at the core of professional development.

It is believed that unclear definitions and setbacks in the differentiation between internships and supervised internships contribute to the context highlighted, negatively impacting student education, as they hinder the development of skills intertwined with the real world of work. Another weakness identified concerns the deficiencies in internships in non-hospital settings. This fact raises concerns about the hegemony of hospital-centered and curative training. It is essential to enhance internships in extra-hospital settings, from an interdisciplinary and broad perspective that values the individual and the community, promoting comprehensive care, which will also contribute to strengthening the SUS.

### Study limitations

Study limitations were related to the documentary data collection, which limits understanding from the actors involved in training, as well as the number of CCPs identified, given the number of courses offered. Further research is suggested, using other approaches and methodologies that can expand this area of knowledge and spark reflections on vocational nursing training in the network.

### Contributions

This research presents contributions aimed at expanding a sphere that is still little explored, in addition to providing support for reflections, improvements and adjustments in the understanding of training in these spaces, favoring the development of more solid educational processes that are coherent with human resources training policies in health and nursing, resulting in improvements in the quality of courses and, consequently, in the healthcare of the population.

## FINAL CONSIDERATIONS

The organizational and pedagogical characterization of VT in nursing in the Federal Network of Professional, Scientific, and Technological Education, based on CCPs, revealed that vocational courses in nursing have structural and organizational specificities, with noticeable convergences and weaknesses. The convergent aspects encompass the intention to respond to social demands, the commitment to quality training, the focus on the use of active methodologies, and the use of qualitative assessment concepts. However, the weaknesses involve discrepancies in meeting project design requirements, the gap between training intention and practice, and the incipient focus on the SUS. Given this context, the challenge of rethinking Pedagogical Projects emerges, requiring adjustments and updates to align them with current demands. From this perspective, it is essential that the Federal Network of Professional, Scientific, and Technological Education explicitly commit to training for the SUS.

The Network presents itself as a strategic model to reiterate the federal government’s commitment to health education. However, to achieve its purpose as a consistent national project, reflections and actions are required that address the challenge of transforming potential into effective action. This requires monitoring the intentionality of education, monitoring, and assessment of the educational process.

## Data Availability

The research data are available only upon request.
