# Supplementary material for: Organizational and pedagogical characterization of vocational training in nursing in the Federal Education Network
Source: Rev Bras Enferm. 2026 Jun 12;79:e20250144. doi: 10.1590/0034-7167-2025-0144 (PMC13262982; doi:10.1590/0034-7167-2025-0144)
Supplement: Translation

# Translation: Caracterização organizacional e pedagógica da formação técnica em enfermagem na Rede Federal de Educação

**DOI:** 10.1590/0034-7167-2025-0144pt

**Authors:** Ludmila Anjos de Jesus, Gilberto Tadeu Reis da Silva, Silvana Lima Vieira, Vânia Marli Schubert Backes

**Affiliations:** I Universidade Federal da Bahia. Salvador, Bahia, Brasil; II Universidade do Estado da Bahia. Salvador, Bahia, Brasil; III Universidade Federal de Santa Catarina. Florianópolis, Santa Catarina, Brasil

**Keywords:** Educação Técnica em Enfermagem, Formação Profissional, Educação em Enfermagem, Formação Profissional em Saúde, Pesquisa em Educação em Enfermagem.

## Abstract

**Objetivos:**

conhecer as características organizacionais e pedagógicas dos cursos de formação técnica de nível médio em enfermagem ofertados pela Rede Federal de Educação Profissional, Científica e Tecnológica.

**Métodos:**

pesquisa descritiva-exploratória, com abordagem qualitativa e natureza documental. Como fonte de dados, utilizaram-se os Projetos Pedagógicos dos Cursos, empregando-se roteiros estruturados. Os dados foram submetidos à análise do conteúdo.

**Resultados:**

foram analisados 26 projetos. Identificaram-se aspectos convergentes quanto à intencionalidade de uma formação de qualidade, imbricada socialmente e com valorização de metodologias ativas. As fragilidades tangenciam discrepâncias no cumprimento das exigências referentes à composição dos projetos, descompassos entre as pretensões formativas e a prática, inconsistências relativas aos estágios, e enfoque incipiente quanto ao Sistema Único de Saúde.

**Considerações Finais:**

os cursos carregam especificidades estruturais e organizativas, emergindo o desafio de fomentar ajustes e atualizações para qualificar a formação, evocando-se o papel estratégico da Rede para transformar a potência em ação efetiva.

## INTRODUÇÃO

A formação em saúde se apresenta como um desafio histórico e contínuo, uma vez que as necessidades de saúde se transformam e se expandem continuamente de acordo com alterações nos contextos sociopolítico-culturais, requerendo, em paralelo, permanentes reavaliações e reorientações das práticas educativas. Sob essa ótica, ressalta-se que a educação profissional se refere a processos que visam à aquisição de conhecimentos e habilidades para o exercício de um ofício, ocupação ou profissão, favorecendo a inserção do indivíduo no mercado de trabalho de forma célere^(1,2)^.

Historicamente, foi desenhada por contornos da dualidade estrutural, que estabeleciam percursos formativos distintos de acordo com a classe social, resultando em atos educativos voltados para as funções intelectuais para as classes socioeconomicamente mais favorecidas e uma preparação instrumental-processual para as classes menos favorecidas, por considerarem o trabalho manual sem mérito social^(3)^. Assim, tradicionalmente, a educação profissional técnica (EPT) se situou delegada às classes menos favorecidas socioeconomicamente, com intenso estímulo ao fazer em detrimento da capacidade de crítica ao conteúdo^(4)^.

Nesse âmbito, a educação profissional de nível médio se delineou com notáveis relevos de grupos hegemônicos, caracterizada pela ênfase no conteúdo e restrição à operacionalização de procedimentos^(2)^. Essa ideologia hegemônica refletiu também na área da saúde, sobretudo nos cursos de formação técnica em enfermagem, que exprimem em sua história questões políticas e socioeconômicas.

O curso de formação técnica em enfermagem, no Brasil, surgiu em 1966, impulsionado pelo quantitativo insuficiente de enfermeiras para atender às necessidades da população e do mercado^(5)^. No atual modelo de organização de prestação de cuidados à saúde, o técnico de enfermagem (TE) desempenha grande parte das atividades de cuidado direto ao paciente, além de representar a maior força de trabalho na saúde, correspondendo a 59,98% dos profissionais de enfermagem^(6)^. Por conseguinte, necessita de uma formação consistente, com uma estrutura sólida pautada em habilidades e competências para atender com qualidade às necessidades de saúde da população, em conformidade com o Sistema Único de Saúde (SUS)^(7)^.

Contudo, no que concerne às diretivas formativas, verifica-se que a prática educativa tem sido guiada pela Lei de Diretrizes e Bases da Educação Nacional (LDB) e pela Resolução n° 1 do Conselho Nacional de Educação, que determina as diretrizes curriculares nacionais para a educação profissional e tecnológica, balizadas pela Lei do Exercício Profissional, em decorrência da lacuna histórica de diretrizes específicas^(8)^. Ressalta-se que, embora essas normativas preconizem a formação integral dos educandos e o desenvolvimento de competências socioafetivas, psicomotoras e cognitivas, vislumbrando-se preparar os sujeitos para o pleno exercício da cidadania no mundo do trabalho e na prática social^(9,10)^, ainda existem escolas onde predomina o interesse de atender às exigências pontuais do mercado de trabalho, sobretudo diante do processo de mercantilização da educação^(4)^.

Nesse sentido, antagonizando a visão estritamente instrumental da educação profissional, a implantação da Rede Federal de Educação Profissional, Científica e Tecnológica, em 2008, buscou interromper a lógica da EPT a serviço da acumulação capitalista, vislumbrando, no trabalho educativo, um importante instrumento de política social, gerador de oportunidades e redutor de desigualdades^(11)^.

Todavia, neste cenário, apesar do papel ordenador da esfera federal na formação de recursos humanos na área da saúde, preconizado na Lei n^o^ 8.080^(12)^, ainda é perceptível a predominância da rede privada na concentração da EPT de nível médio em enfermagem^(13)^, o que acentua o papel potencial da Rede de transformar essa realidade diante da sua função social. São importantes, então, reflexões sobre como ocorre a formação técnica de nível em enfermagem nesse espaço.

Nessa direção, delineou-se a seguinte questão de investigação: como ocorre a formação profissional técnica em enfermagem ofertada pela Rede Federal de Educação Profissional, Científica e Tecnológica?

Considerando que o percurso formativo impacta fortemente o perfil profissional e que a formação se caracteriza por um processo complexo e dinâmico, surgiu a relevância desta temática, tendo em vista as crescentes exigências quanto às competências profissionais e a necessidade cada vez mais pungente de profissionais críticos. Além disso, esta investigação foi desenvolvida em uma área prioritária do Ministério da Saúde, referente à “gestão do trabalho e educação em saúde”^(14)^, viabilizando diálogos entre as políticas de saúde e educação.

Por consistir em uma temática pouco investigada, tendo em vista a escassez de estudos existentes^(15)^, ao passo que se reiteram a originalidade e o ineditismo da proposta, espera-se contribuir para ampliação dessa área do conhecimento, impulsionar novos estudos e favorecer a compreensão de elementos para subsidiar reflexões e ajustes direcionados para melhorias nos processos formativos.

### Relevância do estudo

Este artigo é produto de dissertação de mestrado intitulada “Formação Profissional Técnica em Enfermagem na Rede Federal de Educação Profissional, Científica e Tecnológica”^(16)^, depositada no repositório institucional da Universidade Federal da Bahia (https://www.repositorio.ufba.br/handle/ri/37326).

## OBJETIVOS

Conhecer as características organizacionais e pedagógicas dos cursos de formação técnica de nível médio em enfermagem ofertados pela Rede Federal de Educação Profissional, Científica e Tecnológica.

## MÉTODOS

### Aspectos éticos

Por se tratar de estudo documental, através de materiais de acesso público disponíveis nos sítios virtuais institucionais, foi dispensada a submissão em um Comitê de Ética em Pesquisa. Todavia, os pesquisadores asseguraram todas as questões éticas. Para garantir o anonimato relativo às instituições de origem dos Projetos Pedagógicos, cada projeto recebeu um código composto por uma sigla, cognominada PPC, que significa Projeto Pedagógico do Curso, seguida de um algarismo arábico, como PPC1.

### Tipo de estudo

Pesquisa descritiva de caráter exploratório, com abordagem qualitativa e natureza documental.

### Cenário do estudo

Esta pesquisa apresenta como cenário a Rede Federal de Educação Profissional, Científica e Tecnológica brasileira, composta por instituições de educação superior, básica e profissional, pluricurriculares e *multicampi* ^(11)^. Neste estudo, foram selecionadas as instituições e suas respectivas unidades de ensino, que ofertam o curso técnico em enfermagem em qualquer modalidade.

### Fonte de dados

Como fontes de dados, foram utilizados os Projetos Pedagógicos dos Cursos (PPCs). Ressalta-se que o PPC se configura como um documento oficial que apresenta as concepções e pretensões do arquétipo que se pretende formar, revelando os contornos do processo formativo^(17)^.

### Coleta e organização dos dados

Para o levantamento dos dados relacionados à oferta dos cursos, foi utilizada a Plataforma Nilo Peçanha (PNP), que se trata de ambiente virtual de coleta, validação e disseminação das estatísticas oficiais da Rede Federal de Educação Profissional, Científica e Tecnológica^(18)^. Através da PNP, foi possível levantar as instituições, bem como as unidades acadêmicas que oferecem o curso técnico em enfermagem.

Em seguida, foi realizada visita aos portais oficiais de cada instituição, especificamente de cada aba relativa à unidade acadêmica que oferece o curso técnico em enfermagem, para obtenção do PPC. Destaca-se que, por serem instituições *multicampi*, uma mesma instituição poderá ofertar o curso técnico em enfermagem em uma unidade acadêmica ou *campus*, não ofertando-o em outras unidades. Devido à autonomia administrativa e pedagógica, cursos oferecidos em diferentes unidades acadêmicas da mesma instituição observam PPCs distintos.

Foram excluídas as instituições que, apesar de ofertarem o curso técnico em enfermagem, não possuíam os PPCs disponibilizados em seus sites institucionais ou portais oficiais.

Para apoiar a coleta, foram empregados quatro roteiros estruturados elaborados com auxílio do *Microsoft Excel* ^®^, compreendendo os itens relacionados às questões de interesse, conforme exposto no Quadro 1. A coleta de dados ocorreu, por meio de documentação indireta, de janeiro a julho de 2022. Dois investigadores estiveram envolvidos na coleta de dados, ambos com experiência prévia com pesquisas.

**Quadro 1:** Representação sintética das questões de interesse presentes nos roteiros estruturados, Salvador, Bahia, Brasil, 2025

Identificação do roteiro	Questões de interesse
Roteiro I	Instituições que ofertam o curso Localização Número de cursos técnico em enfermagem ofertados
Roteiro II	Modalidade do curso Modalidade de ensino
Roteiro III	Ano de elaboração do Projeto Pedagógico Equipe de elaboração do Projeto Pedagógico
Roteiro IV	Identificação do curso Justificativa do curso Objetivos Requisitos e formas de acesso Perfil profissional de conclusão Organização curricular Critérios e procedimentos de avaliação de aprendizagem Infraestrutura disponível Perfil de qualificação dos professores e técnico administrativos Certificados e diplomas a serem emitidos Prazo máximo para integralização do curso Identificação das atividades de estágio supervisionado obrigatório

### Análise dos dados

Compuseram este estudo os cursos técnicos em enfermagem oferecidos pela Rede Federal de Educação Profissional, Científica e Tecnológica, independentemente da modalidade de oferta, que disponibilizavam seus PPCs nos sítios oficiais das instituições.

Os dados foram organizados em um banco criado com os recursos do programa *Microsoft Excel* ^®^. Para análise, foi empregada a análise do conteúdo^(19)^. Dessa forma, inicialmente, foi feita a pré-análise, na qual ocorreu o primeiro contato com o campo do *corpus* da pesquisa, mediante a leitura “flutuante” e organização do material, seguida da exploração do material, para posterior tratamento dos resultados, através das interpretações e inferências^(19)^. Os dados foram organizados de acordo com a similitude temática, para posterior interpretação e inferências. Para conferir validade ao estudo, os dados obtidos foram discutidos à luz das legislações e diretrizes nacionais, e das concepções da Rede Federal de Educação Profissional, Científica e Tecnológica.

## RESULTADOS

### Caracterização organizacional dos cursos técnicos em enfermagem ofertados pela Rede Federal de Educação Profissional, Científica e Tecnológica

Dos 48 cursos técnicos em enfermagem ofertados pela Rede Federal de Educação Profissional, Científica e Tecnológica, foram localizados apenas 26 PPCs nos portais oficiais das instituições. Quanto ao ano de publicação desses PPCs, foi possível observar que houve uma variação de 2010 a 2022. Entre os profissionais envolvidos na concepção dos PPCs, há hegemonia dos professores, os quais participaram do desenvolvimento de 12 Projetos Pedagógicos, e apenas dois indicam a presença de representantes estudantis na composição da equipe de elaboração. No tocante à modalidade do curso, há predominância da forma subsequente, revelada em 25 documentos.

Todos os PPCs identificados evidenciam a justificativa para a oferta do curso. Percebe-se que a motivação para oferta dos cursos técnicos em enfermagem na Rede Federal de Educação Profissional, Científica e Tecnológica perpassa o compromisso com o atendimento de necessidades sociais, especialmente aquelas relacionadas à carência de profissionais com essa qualificação. Os PPCs convergem quanto ao comprometimento com a necessidade de contribuir para a formação qualificada e imbricada socialmente, voltada a atender às demandas de saúde locais e regionais, além de promover a expansão da oferta do curso técnico em enfermagem pelo setor público, com o intuito de refletir na saúde e qualidade de vida da população.

[...] *justifica-se a oferta do curso técnico em enfermagem* [...], *respondendo a uma demanda do próprio município e região* [...]. (PPC19)

[...] *carência de cursos de formação técnica públicos na área da saúde, sendo as demandas de formação de trabalhadores nessa área atendidas quase exclusivamente pelo setor privado* [...]. (PPC22)

Apesar da afluência e preocupação com as demandas sociais, apenas três PPCs destacam a formação direcionada para a cidadania, e somente quatro PPCs indicam o alinhamento com as concepções do SUS. Quanto aos objetivos gerais, apresentados na totalidade dos PPCs, há confluência, de maneira geral, com o propósito de formar profissionais qualificados, com competências técnico-científicas sólidas e capazes de contribuir para melhorias na assistência à saúde e na sociedade.

*Formar profissionais-cidadãos qualificados a atuar e intervir no mundo do trabalho, desenvolvendo atividades em diferentes cenários de cuidado da equipe de enfermagem, com ênfase na promoção da saúde* [...]. (PPC1)

[...] *a região é carente de mão de obra qualificada, e a formação destes profissionais proporcionará à comunidade uma melhoria de sua qualidade de vida* [...]. (PPC15)

De maneira geral, os PPCs evidenciados contêm as exigências mínimas referentes à sua composição. Para alguns elementos, sobretudo certificados e diplomas a serem emitidos (PPC2 e PPC10) e requisitos e formas de ingresso (PPC10 e PPC21), houve apenas menção da normativa regulamentadora interna, sem apresentação dos dados.

Todos os PPCs revelam o perfil profissional de conclusão do curso. Nesse âmbito, observou-se o enfoque direcionado para o arquétipo de um técnico em enfermagem crítico reflexivo e capaz de efetuar mudanças em seu meio, com possibilidades de atuação em diversos cenários, conforme exprimem o excerto abaixo:

[...] *deverá ter a capacidade de desenvolver atividades inerentes à sua habilitação, promover uma assistência humanizada de qualidade, capaz de conhecer o processo saúde-doença na sua totalidade, atuando de forma reflexiva, crítica e criativa com o objetivo de atender as necessidades básicas do cliente*. (PPC6)

Constatou-se que a intencionalidade de um perfil profissional afinado com o SUS apareceu de modo tímido nos PPCs, uma vez que apenas sete projetos orbitaram nessa perspectiva. Também foi evidenciada a observância das normativas que orientam a formação e prática dos técnicos em enfermagem, como o Catálogo Nacional dos Cursos Técnicos (CNCT) vigente e a Lei do Exercício Profissional.

[...] *deve compreender os princípios do Sistema Único de Saúde (SUS), valorizando a integralidade e o direito do indivíduo à assistência em qualquer nível da atenção à saúde* [...]. (PPC13)

*O perfil profissional dos egressos do curso técnico em enfermagem está baseado na Lei do Exercício Profissional de Enfermagem nº 7.498/86* [...]. (PPC21)

No tocante à infraestrutura, foram observados pontos sensíveis quanto à apresentação dos dados e recursos disponíveis para oferta do curso técnico em enfermagem na Rede, destacando-se a ausência de laboratórios e a oferta de cursos em condições provisórias.

*A infraestrutura discriminada no PPC sobre o laboratório de enfermagem contemplará a especificidade exigida quando o campus localizar-se na sede definitiva* [...]. (PPC1)

Apesar de a maioria dos PPCs apresentar o perfil de qualificação da equipe, hiatos no perfil de qualificação dos professores e técnico-administrativos, em razão da omissão ou deficiências na descrição detalhada dos dados, foram observados, sobretudo concernentes ao corpo técnico-administrativo. Quanto à qualificação dos docentes, há predominância de mestres e doutores.

### Caracterização pedagógica dos cursos técnicos em enfermagem ofertados pela Rede Federal de Educação Profissional, Científica e Tecnológica

Dos itens relacionados às nuances pedagógicas, pontos frágeis foram verificados no que diz respeito à inexistência de ementas. No tocante aos critérios de avaliação da aprendizagem, foram predominantes processos contínuos, formativos e cumulativos, com enfoque no aspecto qualitativo.

*A avaliação do aproveitamento dos alunos deverá ser formativa, portanto, integral, processual e contínua* [...]. (PPC21)

A indicação do prazo máximo para a integralização do curso foi o que mais apresentou deficiências, já que nove PPCs não abordaram esse ponto. Nos projetos que continham esse tópico, registrou-se uma variação de dois a seis anos como período máximo para conclusão do curso. No tocante à expedição de documentos certificadores após a integralização do curso, verificou-se a hegemonia dos diplomas.

Todos os PPCs continham as organizações curriculares empregadas, sendo evidente a diversificação das estruturas das matrizes, com preponderância da organização em disciplinas e componentes curriculares. Destaca-se que três PPCs (PPC7, PPC8 e PPC12) não revelaram as bibliografias indicadas para a realização do curso. No entanto, dos 15 PPCs que explicitaram as metodologias que fundamentam as organizações curriculares e direcionam o processo de ensino, houve convergência em torno de concepções problematizadoras e interdisciplinares, com valorização das metodologias ativas, da integração e da formação humana.

*O curso técnico em enfermagem adota metodologias de ensino que favorecem o diálogo, o questionamento, a interação e a criatividade* [...]. (PPC12)

[...] *são estratégias didático-pedagógicas adotadas. A articulação e a interdisciplinaridade entre os componentes curriculares e as metodologias integradoras* [...]. (PPC15)

Os PPCs contemplam a descrição da carga horária total (CHt) e distribuída dos cursos, sendo perceptível uma variação de 1.440 horas a 2.724 horas na CHt, com uma média de 1.754 horas. Acerca da distribuição da carga horária, houve uma oscilação de 1.200 a 2.160 horas, relativas às atividades teórico-práticas, e de 260 a 720 horas, concernentes ao estágio supervisionado.

A respeito do estágio supervisionado, observou-se que todos os Projetos Pedagógicos trataram sobre o tema, mas apenas o PPC4 aponta as atividades mínimas a serem realizadas durante o estágio. Houve uma heterogeneidade significativa no que tange ao momento de realização do estágio ao longo do curso e quanto à ocorrência integrada aos componentes curriculares ou de forma independente. Alguns PPCs expressaram o estágio de modo congênere com as práticas dos componentes curriculares e de laboratório. Em relação aos campos de prática, foi predominante o contexto hospitalar.

## DISCUSSÃO

O PPC consiste em um documento basilar que direciona o desenvolvimento dos processos educativos^(20)^. Por ser um documento norteador da formação, preconiza-se que tenha natureza pública, sobretudo quando se tratar de instituições governamentais. Assim, o reduzido número de PPCs disponíveis nos portais oficiais evidencia fragilidades no princípio da transparência dos órgãos públicos. Além disso, inviabiliza a aproximação da sociedade das premissas educacionais e dos propósitos delineados pelas instituições, o que prejudica a dinâmica de interação entre a sociedade e a escola. Notou-se que a maioria dos PPCs apresentava um lapso temporal considerável, e, por isso, são tipificados como defasados. Acentua-se que cabe às instituições a revisão de seu Projeto Pedagógico para mantê-lo como um instrumento de referência atualizado e compatível com as necessidades reais^(10)^.

Quanto às fragilidades evidenciadas na construção dos projetos, ressalta-se que, a partir da gestão democrática dos processos educativos estabelecida pela LDB, infere-se que a elaboração dos PPCs deve se pautar no pluralismo de ideias, abrangendo a comunidade escolar e local para além do âmbito docente^(9)^. A prática de construção do PPC deve ocorrer pautada no diálogo entre todos os atores escolares, no intuito de que o documento retrate os anseios e ideais da comunidade^(21)^.

No que concerne à predominância da modalidade subsequente, depreende-se que é resultante da necessidade de agregar estudantes, sobretudo aqueles em condições de maior vulnerabilidade socioeconômica, que precisam estar inseridos no mercado de trabalho no turno oposto. Além disso, pontua-se que esses cursos geram também um custo menor para as instituições. Contudo, ainda nessa questão, destaca-se que a Rede Federal de Educação Profissional, Científica e Tecnológica tem como princípio a priorização de cursos técnicos, especialmente na forma integrada^(22)^.

Os PPCs identificados cumprem as exigências atinentes à apresentação das justificativas e dos objetivos dos cursos^(10)^. Esse dado demonstra, além da observância de uma normativa, um meio de clarificar a intencionalidade da formação, oferecendo à comunidade parâmetros que norteiam e fundamentam a oferta do curso.

As justificativas tangenciam nuances alusivas às necessidades sociais, o que reforça o compromisso da Rede Federal de Educação Profissional, Científica e Tecnológica com a sociedade e com o desenvolvimento local e regional, além de interferir na oferta predominante do curso técnico em enfermagem pelo setor privado. Cabe ressaltar que a hegemonia do setor privado na oferta do curso técnico em enfermagem^(23)^, muitas vezes, resulta em uma formação fragilizada e atrelada aos interesses de mercado. Assim, a Rede Federal de Educação Profissional, Científica e Tecnológica se coloca como projeto com potencialidade para remodelar esse panorama. Todavia, pondera-se que a oferta do curso técnico de enfermagem ainda é restrita diante de sua abrangência^(24)^. Logo, apesar da demanda, presume-se que o curso não é considerado de forma prioritária na Rede^(23,24)^, o que poderia enriquecer as contribuições para a sociedade.

Por outro lado, embora as justificativas dos cursos estejam pautadas nas demandas sociais, poucos PPCs enfatizam a formação voltada para o exercício da cidadania. Desse modo, considerando o cerne da Rede e a incipiência relacionada à ênfase na cidadania, emerge a necessidade de aproximar os PPCs da formação cidadã e emancipatória, diante da educação como um elemento indispensável para construção e exercício da cidadania. A formação cidadã, além de adquirir maior visibilidade nos Projetos Pedagógicos dos cursos técnicos de enfermagem da Rede, precisa transversalizar o processo educativo, a fim de que não se configure como um discurso superficial e esvaziado.

Constatou-se, claramente, no tocante às justificativas e objetivos, uma tímida expressão no que tange à consonância entre a formação ofertada pela Rede e os princípios do SUS, o que é contraditório, visto que a formação para o SUS se encontra integrada às demandas sociais e de saúde. Nesse contexto, fica evidente que a formação deve estar afinada com o SUS, com o intuito de corresponder a uma resposta efetiva para as necessidades de saúde em diversos cenários em relação aos processos de saúde-doença^(25,26)^. Vale salientar que a Rede, por situar-se na esfera pública federal, compreende um compromisso ainda mais pungente com a formação para o SUS. Nesse sentido, em 2024, foi publicado o Caderno de Diretrizes e Orientações para a Formação Técnica em Enfermagem, o qual intenciona e defende que a formação seja organizada em eixos estruturantes que objetivam o desenvolvimento de competências voltadas para atuação no SUS^(27)^.

Este estudo demonstrou que a Rede Federal de Educação Profissional, Científica e Tecnológica intenciona a formação de um TE capaz de desenvolver as atividades inerentes à sua habilitação e adicionalmente crítico, ético e qualificado, para ser um agente transformador de sua realidade, com múltiplas possibilidades de campos de atuação. Também foi evidenciada a observância das normativas que orientam a formação e prática dos técnicos em enfermagem, como o CNCT e a Lei do Exercício Profissional. Através dos dados obtidos, constatou-se a existência de uma correlação entre o perfil profissional do concluinte técnico em enfermagem e o desenvolvimento de competências envolvendo as dimensões do saber (saber, saber-ser, saber-fazer, saber-conviver), de forma análoga a outro estudo^(21)^.

A ausência de laboratórios de enfermagem e a oferta em condições provisórias, verificadas em alguns PPCs, destoam do que é requerido, podendo constituir um fator complicador na formação. Entretanto, cabe refletir sobre os motivos que podem contribuir para o não cumprimento do que é exigido, especialmente no que concerne às questões financeiras e estruturais das intuições públicas.

Deficiências de dados no que tange ao perfil da equipe, sobretudo os técnicos-administrativos, corroboram para a invisibilização de profissionais, que contribuem para condições e recursos inerentes à dinâmica da formação, ainda que fora da sala de aula^(28)^. A hegemonia de professores mestres e doutores pressupõe um melhor perfil formativo, e alinha-se com a LDB, a qual defende, como vias para qualificação docente, os cursos de mestrado e doutorado^(9)^.

Fragilidades referentes à ausência de ementas e da indicação do tempo para integralização do curso dificultam a compreensão do percurso formativo. Nesse quesito, acresce-se a supressão das bibliografias, divergindo das exigências legais, e dificultando o alcance e entendimento das bases científicas e teóricas que guiam a formação^(10)^. A avaliação da aprendizagem se apresenta em conformidade com a LDB^(9)^. Destarte, a avaliação, valorizada como processual, requer intencionalidade e se encontra intimamente interligada ao sistema de ensino-aprendizagem, devendo transversalizar a formação, distanciando-se de práticas pontuais, isoladas, autoritárias e, muitas vezes, punitivas.

Quanto às organizações curriculares, ressalta-se que disciplinas e componentes curriculares expressam interpretações distintas. Os componentes curriculares abrangem todos os elementos que compõem a integração curricular, sejam eles de natureza teórica ou prática. Já as disciplinas consistem em um conjunto sistematicamente organizado de conhecimentos sobre um objeto, representando um tipo de componente curricular^(29)^. Destaca-se que, muitas vezes, os currículos, quando organizados por disciplinas, segmentam o conteúdo em blocos separados e descontextualizados, sendo comuns na área da saúde blocos básicos e clínicos isolados, o que é preocupante, sobretudo na formação em técnica em enfermagem^(30)^.

A partir do quantitativo considerável de currículos estruturados em disciplinas, pode-se inferir que, quando não associados a estratégias efetivas de integração, denotam a perpetuação de modelos tradicionais marcados pela fragmentação do conteúdo, que divergem das concepções educativas da Rede Federal de Educação Profissional, Científica e Tecnológica. Contudo, ainda nessa seara, ao analisar as metodologias adotadas, foram preeminentes as com características problematizadoras e interdisciplinares com ênfase nas metodologias ativas, as quais convergem com os princípios da formação na Rede Federal de Educação Profissional, Científica e Tecnológica. Tais metodologias se apresentam como uma antítese ao conhecimento disciplinar e fragmentado. Assim, infere-se, a partir desses achados, que a problematização e a interdisciplinaridade se traduzem em iniciativas voltadas para amenizar o descompasso diante da hegemonia de currículos com foco disciplinar.

No tocante à carga horária dos cursos, foi constatada uma polarização importante. Todos os cursos respeitam a carga horária teórica mínima estabelecida, correspondente a 1.200 horas, e grande parte dos cursos ancora a carga horária teórico-prática no mínimo que é determinado pelo CNCT^(31)^. No entanto, fragilidades críticas foram evidenciadas no que tange à carga horária do estágio, favorecidas pela ausência da determinação de uma carga horária mínima para sua realização^(31)^. Todavia, diante do vácuo provocado pela ausência de uma normativa que defina claramente a carga horária mínima exigida para o estágio na formação técnica em enfermagem, o Parecer nº 1/2019 do Conselho Federal de Enfermagem recomenda a adoção de, pelo menos, 400 horas para o estágio curricular para habilitação técnica em enfermagem no país^(32)^. Ressalta-se que a ausência histórica de diretivas específicas para formação técnica em enfermagem forneceu margem para um cenário heterogêneo, com possibilidades concretas de afetar o desenvolvimento adequado do estudante para o alcance do perfil desejado, sobretudo quando admitem restrições alusivas ao contato do discente com situações reais de trabalho.

Foi observada, em alguns documentos, a equiparação entre práticas da disciplina e estágio, ocasionando, inclusive, a contagem da prática em laboratório como atividade de estágio supervisionado. Tal achado demonstra uma importante fragilidade no processo formativo dentro da Rede, dado que a aproximação com situações reais de trabalho se manifesta como o cerne da formação profissional.

Acredita-se que indefinições e retrocessos na diferenciação entre práticas e estágio supervisionado contribuam para o contexto evidenciado, repercutindo em prejuízos na formação do estudante, visto que inviabilizam o desenvolvimento de competências imbricadas com o mundo real do trabalho. Outro ponto frágil verificado diz respeito às deficiências de realização de estágio em contextos não hospitalares. Este fato resgata reflexões sobre a hegemonia de uma formação hospitalocêntrica e curativista. Torna-se fundamental potencializar a realização de estágios em espaços extra-hospitalares, sob a perspectiva interdisciplinar e ampla, que valorize o indivíduo e a coletividade, em prol da integralidade da assistência, o que também contribuirá para o fortalecimento do SUS.

### Limitações do estudo

As limitações do estudo foram relacionadas à coleta de dados, realizada de forma documental, o que restringe a compreensão a partir dos atores envolvidos na formação, assim como o número de PPCs identificados, diante do número de cursos ofertados. Sugere-se a realização de novas pesquisas, com a utilização de outras abordagens e metodologias que possam ampliar essa área do conhecimento e suscitar reflexões acerca da formação técnica em enfermagem na Rede.

### Contribuições

Esta pesquisa apresenta contribuições voltadas à ampliação de uma esfera ainda pouco explorada, além de fornecer subsídios para reflexões, aperfeiçoamentos e ajustes na compreensão da formação nesses espaços, favorecendo o desenvolvimento de processos educativos mais sólidos e coerentes com as políticas de formação de recursos humanos nas áreas da saúde e enfermagem, repercutindo em melhorias na qualidade dos cursos e, consequentemente, no cuidado à saúde da população.

## CONSIDERAÇÕES FINAIS

A caracterização organizacional e pedagógica da formação técnica em enfermagem na Rede Federal de Educação Profissional, Científica e Tecnológica a partir dos PPCs evidenciou que os cursos técnicos em enfermagem carregam especificidades estruturais e organizativas, sendo perceptíveis convergências e fragilidades. Os aspectos convergentes perpassam a intencionalidade de responder às demandas sociais, ao comprometimento com uma formação de qualidade, ao enfoque na utilização de metodologias ativas e ao emprego de concepções qualitativas da avaliação. Já as fragilidades tangenciam discrepâncias no cumprimento das exigências referentes à composição dos projetos, ao distanciamento entre a intencionalidade da formação e a prática, e ao enfoque incipiente quanto ao SUS. Diante desse contexto, emerge o desafio de repensar os Projetos Pedagógicos, sendo relevantes adequações e atualizações para compatibilizá-los com as demandas atuais. Nessa perspectiva, torna-se essencial que a Rede Federal de Educação Profissional, Científica e Tecnológica assuma explicitamente o compromisso com a formação para o SUS.

A Rede se apresenta como modelo estratégico para reiterar o compromisso da esfera federal com a formação em saúde. Todavia, para concretização de sua finalidade como projeto nacional consistente, são necessárias reflexões e ações que atravessam o desafio de transformar a potência em ação efetiva, sendo necessários a vigilância da intencionalidade da formação, o monitoramento, e a avaliação no e do processo formativo.

## Data Availability

Os dados de pesquisa só estão disponíveis mediante solicitação.
